# Stable Spirocyclic Meisenheimer Complexes

**DOI:** 10.3390/molecules13061282

**Published:** 2008-06-09

**Authors:** Rabih O. Al-Kaysi, Iluminada Gallardo, Gonzalo Guirado

**Affiliations:** 1 Department of Basic Sciences, King Saud Bin Abdulaziz University for Health Sciences-National Guard Health Affairs. Building Mail Code 3124. Riyadh 11423. Kingdom of Saudi Arabia; Phone: (966)-5-61429528; 2 Departament de Química, Universitat Autònoma de Barcelona, 08193-Bellaterra, Barcelona, Spain; Phone: (+) 00 34 93 581 48 82; Fax: (+) 00 34 93 581 29 20

**Keywords:** Spirocyclic-, Zwitterionic-, Meisenheimer- Complexes, Fluorescent Compounds

## Abstract

Meisenheimer complexes are important intermediates in Nucleophilic Aromatic Substitution Reactions (S_N_Ar). They are formed by the addition of electron rich species to polynitro aromatic compounds or aromatic compounds with strong electron withdrawing groups. It is possible to distinguish two types of Meisenheimer or σ-complexes, the σ^H^-complex or σ^X^-complex (also named ipso), depending on the aromatic ring position attacked by the nucleophile (a non-substituted or substituted one, respectively). Special examples of σ^X^- or ipso-complexes are formed through intermediate spiro adducts, *via* intramolecular S_N_Ar. Some of these spirocyclic Meisenheimer complexes, a type of σ^X^-complex, are exceptionally stable in solution and/or as solids. They can be isolated and characterized using X-ray, and various spectroscopic techniques such as NMR, UV-Vis, IR, and fluorescence. A few of these stable spirocyclic Meisenheimer complexes are zwitterionic and exhibit interesting photophysical and redox properties. We will review recent advances, synthesis and potential applications of these stable spirocyclic Meisenheimer complexes.

## Introduction

**Scheme 1:**
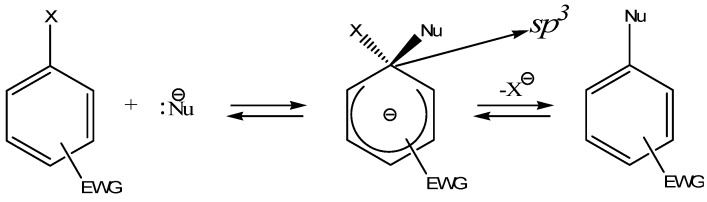
Where X is a good leaving group and EWG an electron withdrawing group.

Nucleophilic aromatic substitution (S_N_Ar) is an important reaction which takes place when electron rich species (nucleophiles) add to an aromatic ring containing electron withdrawing groups [1]. It is believed that this reaction generally proceeds through an addition-elimination mechanism. In the first step the nucleophile would preferably attack the position *ipso* to the electron withdrawing group of the electron deficient aromatic ring to yield an anionic sigma adduct intermediate. Typically; this intermediate with a tetrahedral (sp^3^) carbon is unstable, and the reaction could either proceed forward by rearomatization to generate the substituted product or simply revert back to the reactants (Scheme 1). 

When the aromatic ring is substituted with various electron withdrawing groups (EWG) such as ‑NO_2_, -CN, -CF_3_ or -SO_2_CF_3_, the anionic sigma complex is stabilized due to the added delocalization of the negative charge by the EWG in conjugation with the aromatic ring. This extra stabilization is sometimes sufficient enough for these anionic sigma intermediates to be isolated and characterized using X-ray crystallography, NMR, IR, UV-Vis and fluorescence spectroscopy. These intermediates are also called σ- or Jackson-Meisenheimer-complexes, named after the scientists who proposed a possible quinoid-like structure of the compound formed after adding an equimolar amount of sodium methoxide to an acetone solution of 2,4,6-trinitroanisole [2,3]. The reaction yielded an isolable and stable red colored solid. The intense red color of these complexes was a major contributor to the early interest in these complexes and their use as dyes [4,5,6,7].

**Scheme 2:**
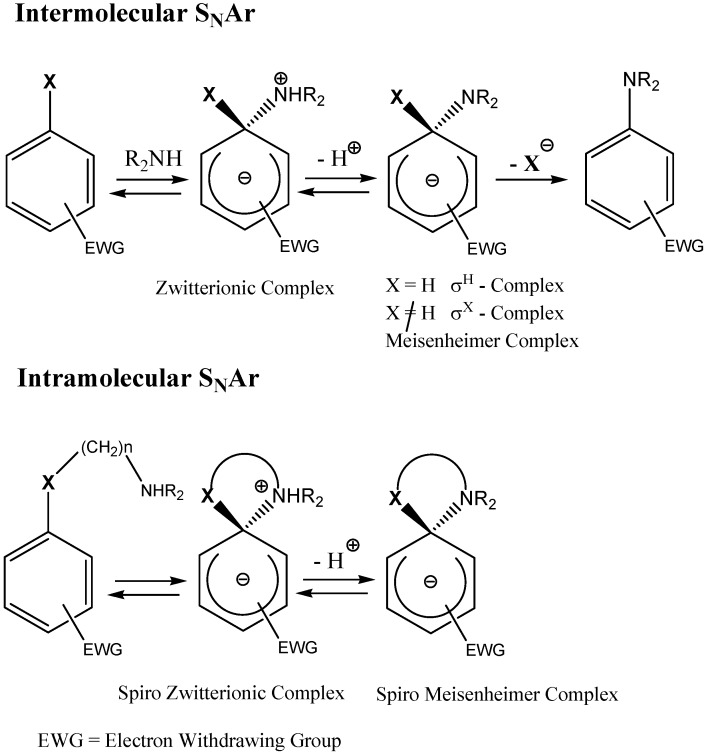


When the electron rich species is a neutral nucleophile, such as an amine or phosphine, the formation of a zwitterionic compound prior to the formation of the σ-complex has been postulated, although; in most cases, this zwitterion has not been isolated. Hence, it is possible to distinguish two types of σ-complexes, the σ^H^-complex or σ^X^-complex (also named ipso), depending on the aromatic ring position attacked by the nucleophile (a non-substituted or substituted one, respectively, Scheme 2, top). Special cases of σ^X^- or ipso-complexes are formed through intermediate spiro adducts, via intramolecular S_N_Ar. These have been unambiguously characterized in several instances (Scheme 2, bottom). It is important to highlight that when a σ^H^-complex is formed the reaction would rarely spontaneously evolve, by elimination of the hydride anion, to afford the substituted product (Scheme 2 top).

**Scheme 3:**
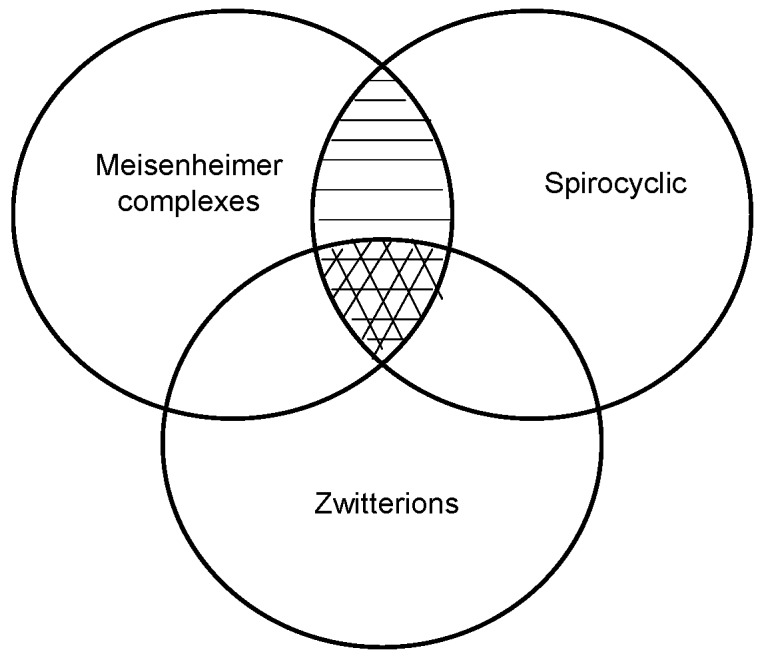


In the last four decades several reviewers have discussed the subject of anionic sigma complexes [8,9]. In this review we will attempt to narrow down this broad subject to cover advances in the synthesis of spirocyclic Meisenheimer complexes (SMCs) and zwitterionic spirocyclic Meisenheimer complexes (ZSMCs). Scheme 3 is a pictorial presentation of what the review will focus on. The lightly shaded area (SMCs) will be reviewed with details of the synthetic approach utilized to obtain these compounds. The heavily shaded area (ZSMCs) will be explicitly discussed and reviewed with emphasis on the synthesis and properties of the most recently reported ZSMC (diagram is not to scale).

## 2. a. Spirocyclic Meisenheimer Complexes (SMCs)

**Scheme 4:**
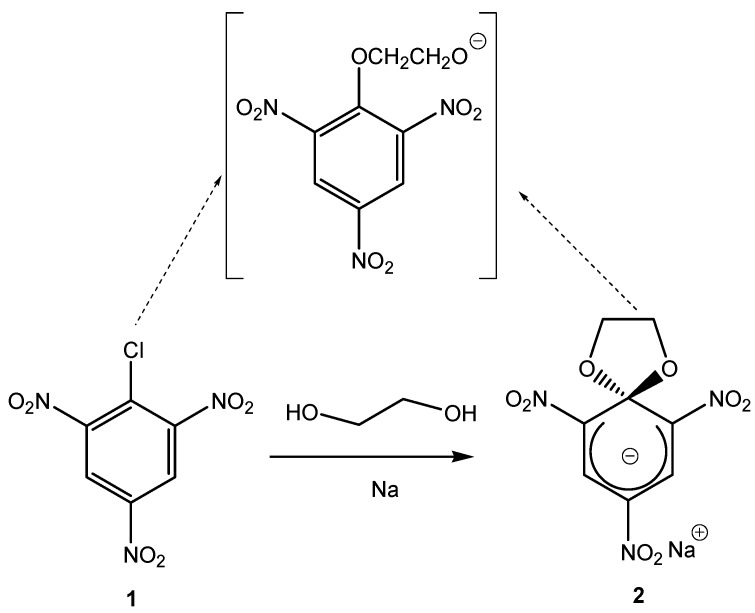


In this section we will discuss advances in the synthesis of stable spirocyclic Meisenheimer complexes produced by the possible formation of intermediate spiro zwitterionic adducts, via intramolecular S_N_Ar reactions (Scheme 2, bottom). We will exclude discussions pertaining to the kinetics of formation of these SMCs. The first spirocyclic Meisenheimer complex was reported in 1965 (Scheme 4) [10]. The reaction of picryl chloride (**1**) with ethylene glycol and two equivalents of Na metal afforded the spirocyclic Meisenheimer complex (**2**) as a sodium salt after an intermolecular nucleophilic aromatic substitution of chlorine (probably by an addition-elimination mechanism, Scheme 2, top), followed by an intramolecular aromatic substitution reaction (Scheme 2, bottom). This is also an example of a σ-complex with oxygen containing nucleophiles. The UV-Vis spectrum of this complex showed two absorption bands (maxima at 414 nm and 490 nm), consistent with an acyclic Meisenheimer complex. Compound (**2**) was stable enough to be isolated after filtering the red colored salt and washing out the excess ethylene glycol with hexane.

**Scheme 5:**
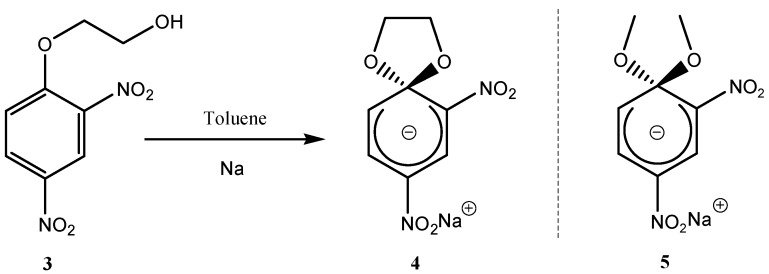


When an equivalent amount of sodium was added to a 2-(2,4-dinitrophenoxy)ethanol (**3**) in toluene solution (Scheme 5), SMC (**4**) was obtained, via an intramolecular S_N_Ar reaction, as a stable sodium salt which was separated and washed with petroleum ether to remove the excess reagents [11]. This compound is analogous to compound (**2**). The synthesis of a related compound (**4**) showed that having two electron withdrawing groups (-NO_2_) at the 2-, and 4-positions of the benzene ring were enough to stabilize the anionic σ-complex formed by the reaction of an oxygen nucleophile. A comparison of the stability of SMC (**4**) and the related acyclic dimethoxy compound (**5**) shows that SMC (**4**) is 50% more stable in acids than (**5**) [12]. This extra stability was attributed to the spirocyclic 1,3-dioxolane ring [13].

**Scheme 6:**
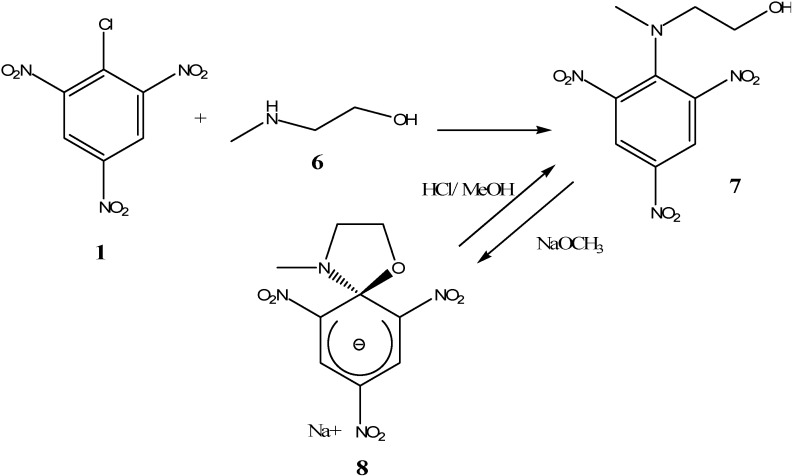


Hydroxylamines can also be used as nucleophiles. The reaction of picryl chloride (**1**) with one equivalent of *N*-methylethanolamine (**6**) in toluene gave the stable *N*-substituted picric acid derivative (**7**). In compound (**6**) the NH-Me group is more nucleophilic than the OH group, thus the nitrogen will first attack the ipso position of the ring. Later the chlorine atom is eliminated via an addition-elimination mechanism. Further treatment of compound (**7**) with sodium methoxide deprotonated the OH group on compound (**7**) and afforded SMC (**8**) as a stable sodium salt (Scheme 6). This is an example of an asymmetric SMC formed by inter- followed by intramolecular attack of two different nucleophiles on the same *ipso* position of the aromatic ring. This SMC is unstable in acidic solutions and reverts back to (**7**) upon treatment with an equivalent amount of HCl in methanol [14].

**Scheme 7:**
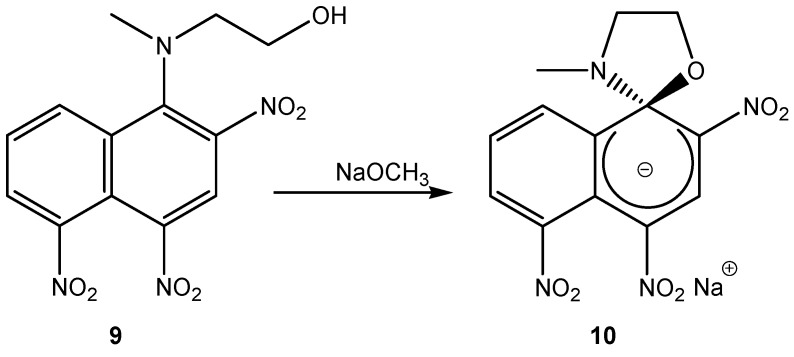


It is interesting to note that the formation of SMCs is not only limited to polynitrobenzene derivatives. The reaction of one equivalent of *N*-methylethanolamine (**6**) with 1-chloro-2,4,5-trinitro-naphthalene to afford compound (**9**), is a good example. When sodium methoxide was added to a solution of (**9**), the reaction yielded the stable SMC (**10**) (Scheme 7) as an isolable red colored salt. Neither compound (**10**) nor the previously mentioned compound (**8**) was stable in acidic solutions or protic solvents [15].

**Scheme 8:**
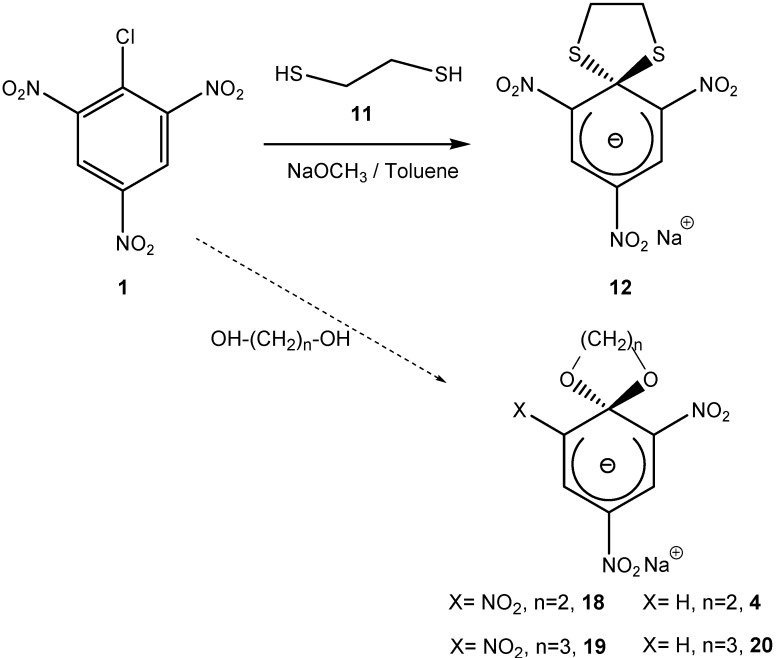


Either the amino or alcohol terminal nucleophile groups can be replaced by thiol groups. The use of sulfur as a nucleophile instead of N or O resulted in another class of SMCs (thio-SMCs). The addition of (**1**) to one equivalent of 1,2-dithioethane (**11**) and two equivalents of NaOCH_3_ led to the dithio-SMC (**12**) as a stable amorphous red-colored sodium salt. The absorption spectrum of (**12**) in DMSO was similar to that of the acyclical thio-Meisenheimer complex (Scheme 8). In both cases the absorption maxima were at 454 nm, 534 nm, and 564 nm. SMC (**12**) was stable in an acidic solution. The opening of the 1,3-dithiolane ring was catalysed by Hg^2+^ [16]. A similar reaction with 1,3-dithiopropane did not afford the spirocyclic Meisenheimer complex [17].

**Scheme 9:**
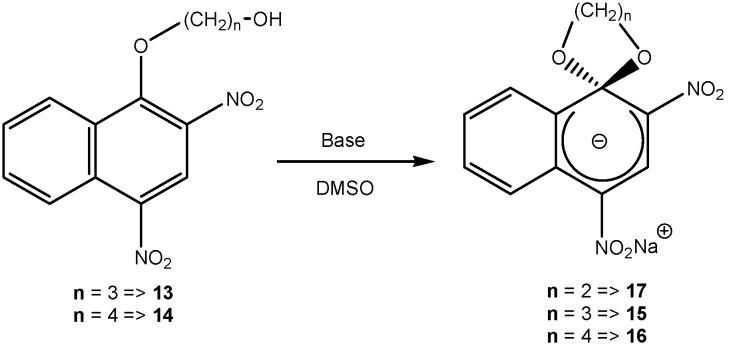


MCs were not only limited to a 5-membered spirocyclic ring attached to the electron deficient aromatic ring. Attempts have been made to expand the 5 member ring to a 6- or 7-membered ring. The reaction of 1-(3-hydroxypropoxy)- (**13**) and 1-(4-hydroxybutoxy)-2,4-dinitronaphthalene (**14**) respectively with one equivalent of a strong base affords the SMCs (**15**) and (**16**), respectively. The yield of SMC formation was compared to that of 5-membered ring SMC (**17**). A larger ring size makes the reaction slower and less efficient, thus the yield of the corresponding SMC is very low. The rate of acid catalyzed ring opening of (**15**), (**16**), and (**17**) did not show any dependence on ring size, which reflects the importance of kinetic rather than thermodynamic factors in the formation of the larger ring sizes [18] (Scheme 9). Similar reaction conditions were used to synthesize the trinitro- and dinitrobenzene SMC derivatives (**4**), (**18**), (**19**), (**20**) (Scheme 8). A trend similar to that observed for the 2,4-dinitronaphthalene derivatives was observed, one difference being that a 4-membered spirocyclic ring (n = 4) could not be formed with either the 2,4-dinitro- or 1,3,5-trinitrobenzene derivatives [19,20,21,22].

**Scheme 10:**
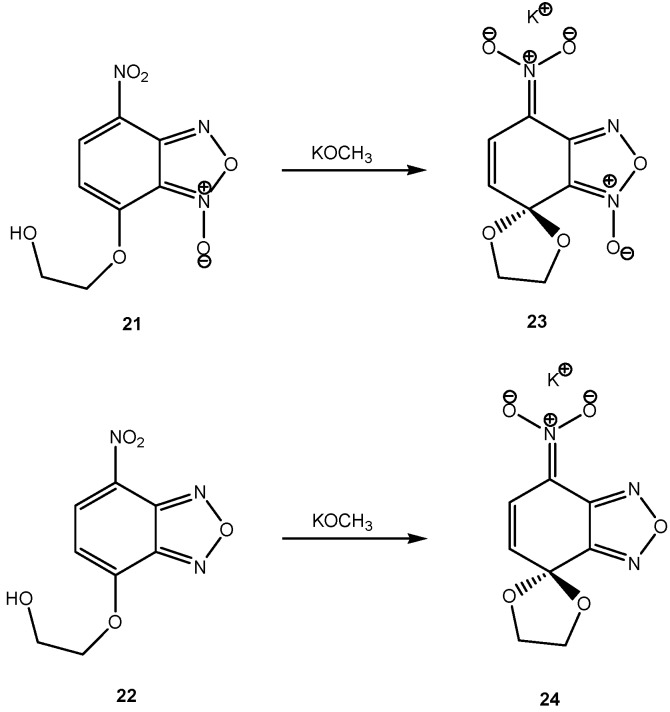


It is worth highlighting that the heterocyclic nitro substituted benzofuroxan- and benzofurazan-1-oxide rings can form stable SMC salts [23]. Intramolecular S_N_Ar reaction of (**21**) and (**22**) in methanol solution of potassium methoxide provided the SMCs (**23**) and (**24**) as stable potassium salts (Scheme 10). Unlike the SMCs derived from polynitrobenzene or naphthalene the absorption spectra of (**23**) and (**24**) were blue shifted and gave two absorption peak maxima at 330 nm and 339 nm, respectively, thus affording colorless salts. The polynitro benzofurazan-1-oxide starting material and some of its acyclic Meisenheimer complexes are unstable and will detonate upon friction when dry [24]. 

**Scheme 11:**
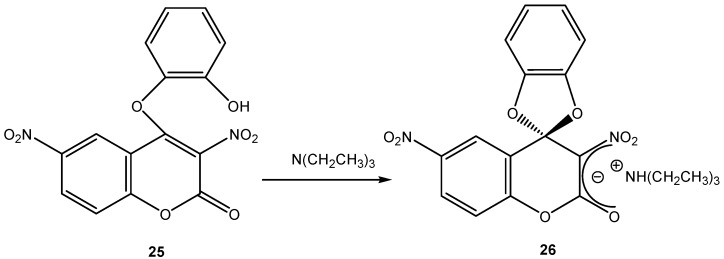


Nitro-substituted benzopyran-2-one (**25**) was also used to synthesize SMCs from a base catalyzed cyclization reaction (Scheme 11). Compound (**26**) is a stable salt of an organic cation. This was possible because the phenolic OH was acidic enough to be deprotonated by a weak base such as triethylamine [25]. Quaternary ammonium organic cations are “softer” than metal cations such as K^+ ^or Na^+^. This will have an effect on the distribution of the negative charge in the Meisenheimer complex.

## 2. b. Russian contributions to SMCs

During the past four decades, Russian chemists were very interested in synthesizing novel Meisenheimer complexes. The advent of ^1^H- and ^13^C-NMR made it easier to study, analyze and characterize complex SMCs. Victor N. Knyazev, V. N. Drozd and co-workers contributed significantly to the field of spirocyclic Meisenheimer complex synthesis. They published over 55 papers, mainly in the *Russian Journal of Organic Chemistry*, that describe the synthesis, properties and kinetics of some novel spirocyclic Meisenheimer complexes. Some of these SMCs were isolated and fully characterized. A representative list of these compounds is displayed in Table 1, with starting materials used and synthesis conditions. 

**Table 1:** List of stable SMCs and reaction conditions.

SMC	Reactants	Conditions	Reference
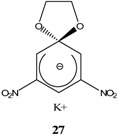	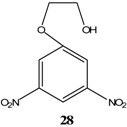	KOCH_3_ / MeOH	[26]
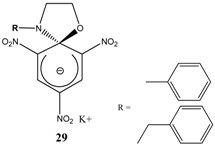	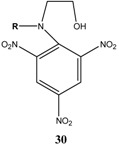	KOCH_3_ / MeOH	[27]
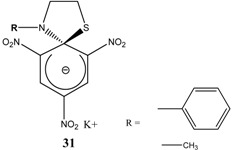	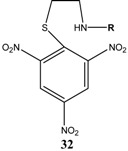	KOC(CH_3_)_3_/ *t*-butanol	[28]
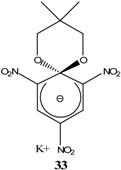	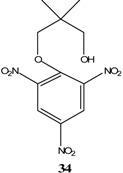	KOC(CH_3_)_3_/ *t*-butanol	[29]
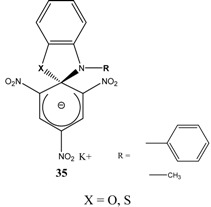	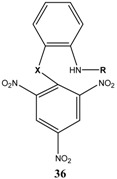	KOC(CH_3_)_3_/ *t*-butanol	[30]
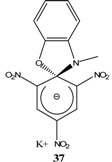	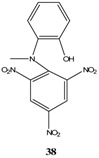	KOC(CH_3_)_3_/ t-butanol	[30, 31]
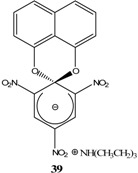	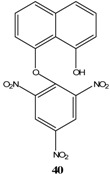	Et_3_N/ CH_2_Cl_2_	[32]
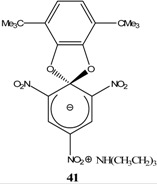	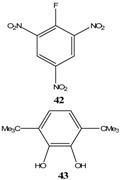	Et_3_N/ CH_2_Cl_2_	[37]
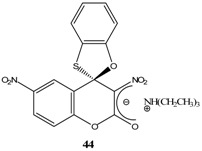	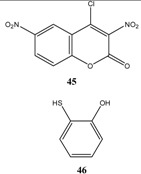	Et_3_N/ CH_2_Cl_2_	[48]
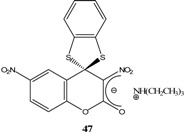	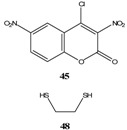	Et_3_N/ CH_2_Cl_2_	[49]
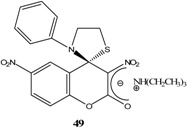	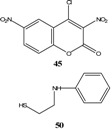	Et_3_N/ CH_2_Cl_2_	[53]

It is worth mentioning that most of the SMCs synthesized by V. N. Knyazev and co-workers revolved around a similar strategy with slight variations in the chemical structures of the reactants [33,34,35,36,38,39,40,41,42,43,44,45,46,47,50,51,52].

## 3. Zwitterionic Spirocyclic Meisenheimer Complexes (ZSMCs)

**Scheme 12:**
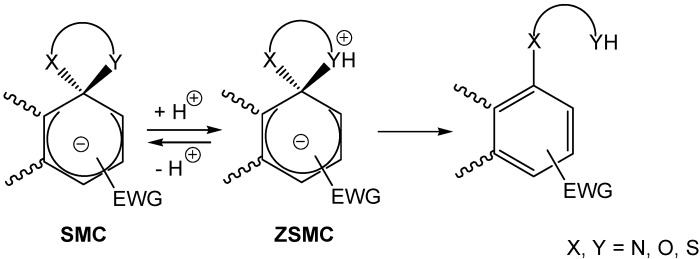


In the previous section we briefly surveyed some of the stable SMCs prepared by intramolecular S_N_Ar attack (Scheme 2, bottom) by a nucleophile. A common feature among all these SMCs was that the anionic sigma complex was balanced out by an independent cation. The cation was either a Na^+^, K^+^, R_4_N^+^, or any other organic or inorganic cation. Salt SMCs have limited solubility in low polarity organic solvents and sometimes the cation interfered in the photophysical and electrochemical properties of the SMC. It is believed that the type of cation complementing the anionic sigma-complex has an effect on the negative charge distribution of the sigma complex and molecular geometry of the σ-complex. An interesting type of SMCs do not contain an independent cation, instead the anionic σ-complex is balanced out by a cation that is chemically attached to the SMC. These interesting compounds, named Zwitterionic Spirocyclic Meisenheimer Complexes (ZSMCs), are only stable in a buffered solution; hence they could rarely be isolated. They were identified by their UV-Vis and proton NMR spectra. Studies of these ZSMCs were in most cases limited to kinetics [55,56,57]. In some rare cases successful isolation of an acyclic ZMC has been reported [54]. Generally, the unstable ZSMCs were prepared by careful protonation of the nucleophile directly attached to the (sp^3^) carbon center of the SMC. It is potentially possible to reversibly form a ZSMC or SMC by protonation and deprotonation reactions on the SMC or ZSMC, respectively. Since ZSMCs are usually unstable intermediates, they would either rearomatize to the acyclic product or loose a proton to generate back the conjugate SMC (Scheme 12). Rearomatization is usually the favored reaction pathway.

**Scheme 13:**
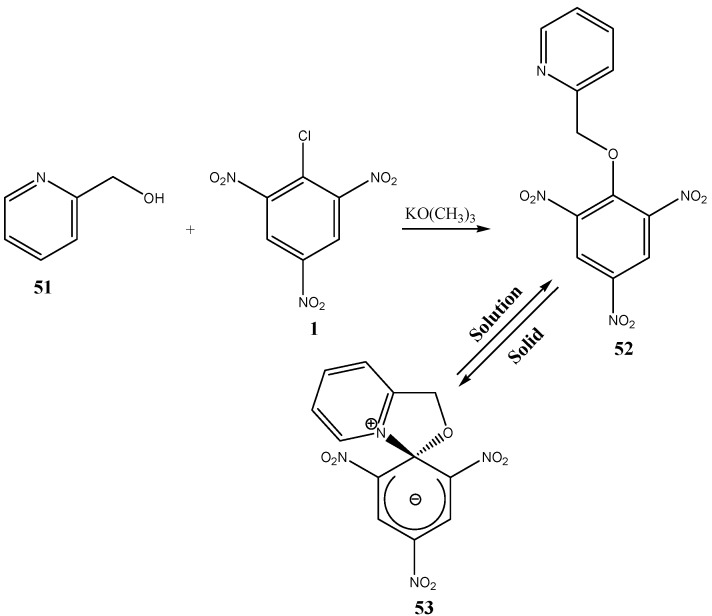


Taking into account the general strategy depicted in Scheme 2, bottom, compound (**51**) was added to (**1**) in dichloromethane in the presence of an equimolar quantity of KOC(CH_3_)_3_ to yield compound (**52**). A solution of (**52**) was found to be in equilibrium with the ZSMC (**53**) (Scheme 13). This became clear when the absorption spectrum of a solution of (**52**) in dichloromethane showed red shifted peaks consistent with a Meisenheimer complex. Also, the H-NMR spectrum of (**52**) showed chemical shifts consistent with structure (**53**) [58]. A crystalline sample of (**52**) existed only in the ZSMC form (**53**). The instability of (**53**) in solution could be attributed to the proximity of the positive charge to the (sp^3^) carbon and the bulkiness of the pyridine group, a feature all too common with unstable ZSMCs formed by protonation of the sp^3^ bonded nucleophile.

**Scheme 14:**
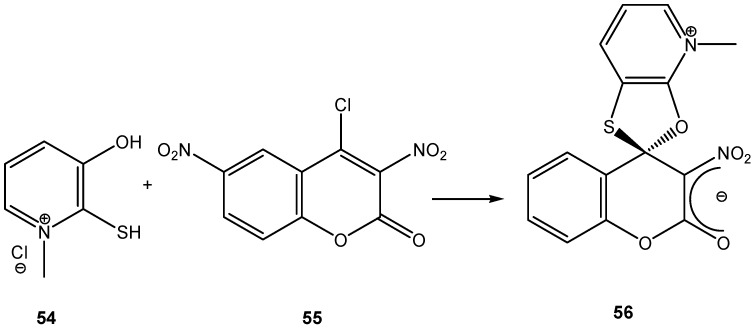


The reaction of (**54**) and (**55**) in toluene in the presence of a base afforded a stable ZSMC (**56**) (Scheme 14). Unlike (**53**), a solution of (**56**) was not in equilibrium with an acyclic complementary molecule. The positive charge was localized on the pyridine ring nitrogen, far from the sp^3^ carbon [59,60].

**Scheme 15:**
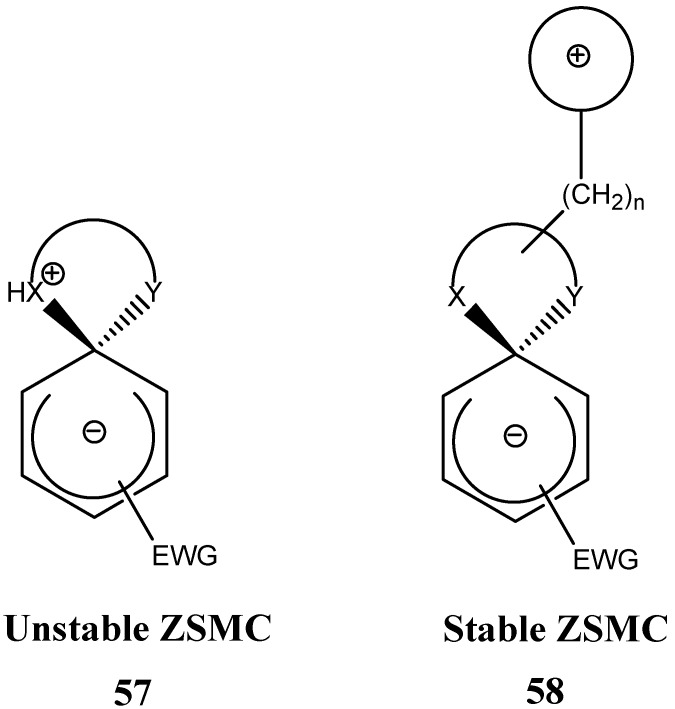


A wide variety of stable ZSMCs were synthesized by adding a nucleophile, attached to an ammonium cation (Compound **59**), to the electron deficient aromatic ring. These ZSMCs are zwitterionic in nature but differ only in that the metal cation is replaced with an organic cation. The solubility of ZSMCs like (**58**) in organic solvents increased significantly relative to (**57**) (Scheme 15).

**Scheme 16:**
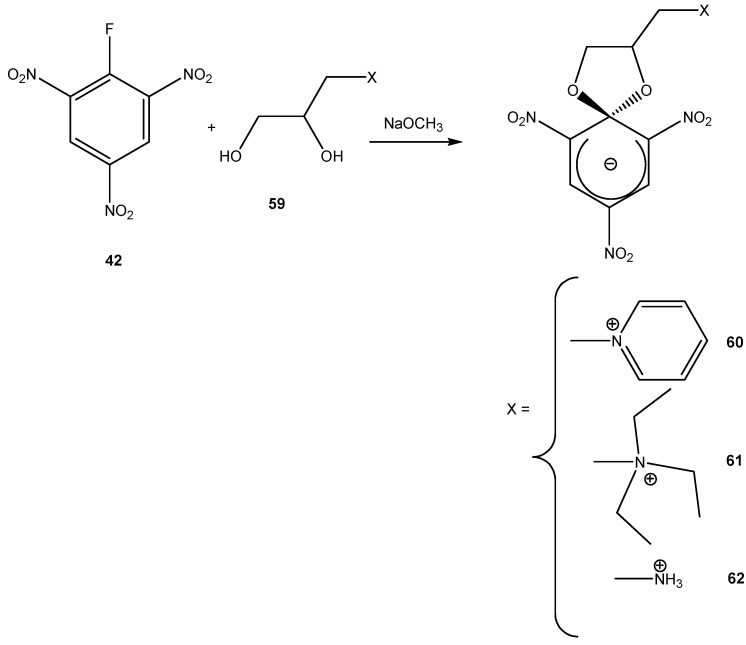


Compounds (**60**), (**61**) and (**62**) were synthesized following a procedure similar to that used for the synthesis of **33**. The product was then extracted with organic solvents to yield the ZSMC (Scheme 16). Since the ZSMC compounds were stable, it was possible to definitively confirm the zwitterionic nature of (**60**), (**61**) and (**62**) [61] by determination of their X-ray structure.

## 4. Fluorescent ZSMCs

**Scheme 17:**
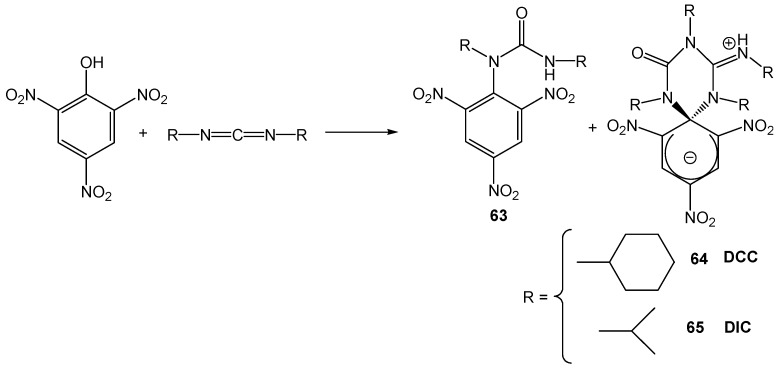


The strategies to synthesize stable ZSMCs involve a multi step synthesis under basic conditions. [62,63,64,65,66,67,68,69,70]. The reaction of picric acid with *N,N'*-dicyclohexylcarbodiimide (DCC) in dichloromethane yields *N*-picrylurea (**63**) as the major product and 1% of a red fluorescent compound (**64**). The reaction was investigated almost four decades ago [62,63,64,65,66], however the product (**64**) was never characterized or properly identified up to now [67]. In fact several papers proposed the wrong structure [68]. In order to increase the yield of the red product synthesis, DCC was replaced by *N,N'*-diisopropylcarbodiimide (DIC). The reaction of picric acid with DIC resulted in 16% of the ZSMC (**65**) [69,70] (Scheme 17).

**Scheme 18:**
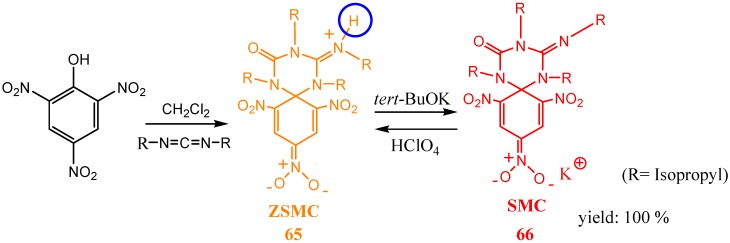
Reproduced with permission from Ref. [71]. Copyright 2006 Elsevier B.V.

Single crystal X-ray structure determination of this compound confirmed its zwitterionic nature and placed the negative charge density on the *para* nitro group [71]. Compound (**65**) is unique in many aspects. It is very stable and exhibits wide solubility in organic solvents of different polarity. It fluoresces in the solid or solution state with a quantum yield of 0.5 in dichloromethane. The fluorescent properties of (**65**) made it suitable as a fluorescent acid base indicator. Adding one equivalent of a base such as *tert-*BuOK abstracts the ammonium hydrogen to yield the nonfluorescent SMC (**66**) [70]. Compound (**66**) was isolated and fully characterized [72]. ZSMC (**65**) could be quantitatively regenerated from (**66)** by addition of one equivalent of perchloric acid (Scheme 18).

**Scheme 19:**
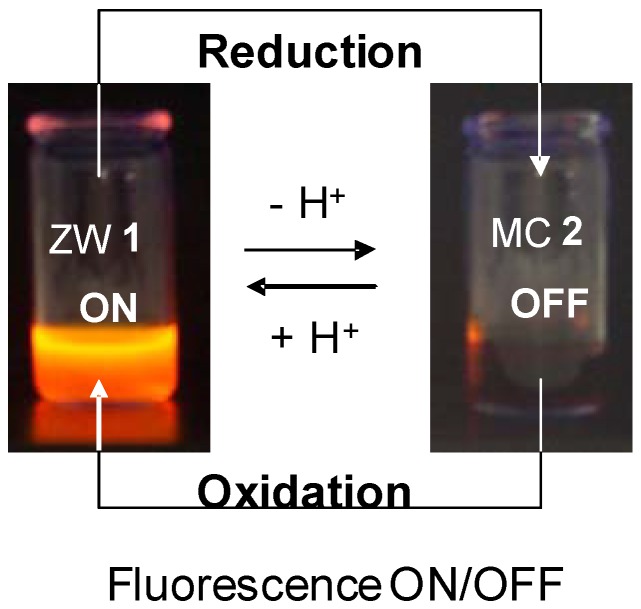
Reproduced with permission from Ref. [70]. Copyright 2006 Elsevier B.V.

Moreover, coordination of other Lewis acids to the guanidine group nitrogen in (**66**), suppresses the quenching mechanism and allows the SMC to fluoresce. The same reversible formation of (**65**) and (**66**) could be achieved through electrochemical oxidation/reduction. The redox properties of both (**65**) and (**66**) have been studied using electrochemical oxidation-reduction mechanism (established by cyclic voltammetry; classical and with ultramicroelectrodes) and controlled-potential electrolysis. A potential fluorescence switching system has been established, since fluorescent properties can be reversibly modulated by a conversion of both states (ON, **65**) and (OFF, **66**) upon reduction of (**65**) or oxidation of (**66**) (Scheme 19). 

Interestingly, spectroelectrochemical joint measurements show that switching of the (**65**–**66**) pair can also be attained electrochemically, thus unraveling the versatile functioning of this system. The ultimate limit of monitoring the reversible on–off operation of individual switch molecules is reached by means of single-molecule fluorescence spectroscopy, which demonstrates the potential of the (**65**–**66**) system to be used as a true single-molecule switch on the nanometer scale [71]. Compound (**65**) was also used to synthesize a clear nanosized suspension of ZSMC in water by using a probe sonicator to “mill by sound” a precipitated tetrahydrofuran solution of (**65**) in water [73]. The nano-suspension does not scatter visible light because the average particle size was close to 70 nm. These aqueous suspensions are very stable and will not precipitate out even after standing for three years at room temperature. Finally, 0.1 molar ZSMC (**65**) was also incorporated into PMMA polymer films. Even under such a high loading concentration the fluorescence of (**65**) was not self quenched. The shapes of the absorption and fluorescence spectra were not perturbed with respect to the spectra of less concentrated samples. This opens up the possibility of using this compound or similar derivatives as a dye and coloring additive in polymers [74].

## 5. Conclusions

We have surveyed the different types of SMCs and ZSMCs and their synthesis. In general SMCs are more stable than ZSMCs and more versatile. The stability of ZSMCs is primarily determined by how far the cation is from the spiro carbon of the Meisenheimer complex. Clever synthetic strategies were developed to synthesize stable ZSMCs based on the cation being sigma bonded to the spirocyclic ring and farther from the spiro center. We have also discussed the synthesis of a novel ZSMC via a one step addition of DIC to picric acid. This ZSMC could be inter-converted between a SMC and ZSMC by adding the appropriate acid or base or by electrochemical oxidation reduction sequences. We have discussed many properties of this ZSMC, for example its unique photophysical properties and the observation that nanoparticle dispersions in water are stable up to 3 years at room temperature.
